# HIV update for the non-HIV specialist and general physician

**DOI:** 10.1016/j.clinme.2026.100628

**Published:** 2026-07-19

**Authors:** Miriam Ringshall, Jaime H. Vera

**Affiliations:** a University Hospitals Sussex NHS Foundation Trust, Eastern Road, Brighton BN2 5BE, UK; b Department of Global Health and Infection, Brighton and Sussex Medical School, University of Sussex, Brighton BN1 9PX, UK

**Keywords:** Antiretroviral therapy, HIV

## Abstract

HIV has become a manageable chronic condition, one of the major successes of modern medicine. People living with HIV can now expect near-normal life expectancy with effective treatment, yet important challenges remain. Comorbidities, prevention of new infections, and the persistence of stigma continue to affect outcomes. Addressing these issues requires coordinated care, with general practice, hospital services and specialist HIV teams working closely together. This article aims to support healthcare professionals by summarising current best practice in treatment, outlining advances in prevention and early diagnosis, and exploring how stigma can be reduced both within healthcare and in the wider community. These efforts are essential if the UK is to meet its goal of ending new HIV transmissions by 2030.

## Highlights

• Effective and tolerable antiretroviral therapies have transformed HIV into a chronic condition.
• Timely HIV testing avoids the clinical consequences of late diagnosis and reduces the time someone can unknowingly pass on HIV.
• U=U (undetectable=untransmittable) means there is zero risk of transmission through sex when the viral load is undetectable.
• Removing barriers to oral and injectable HIV-PrEP access paves the way to zero new transmission by 2030.
• Stigma is a major barrier to testing and engagement in care. Every healthcare interaction can reinforce or reduce stigma.

## Graphical Abstract

**Figure:**
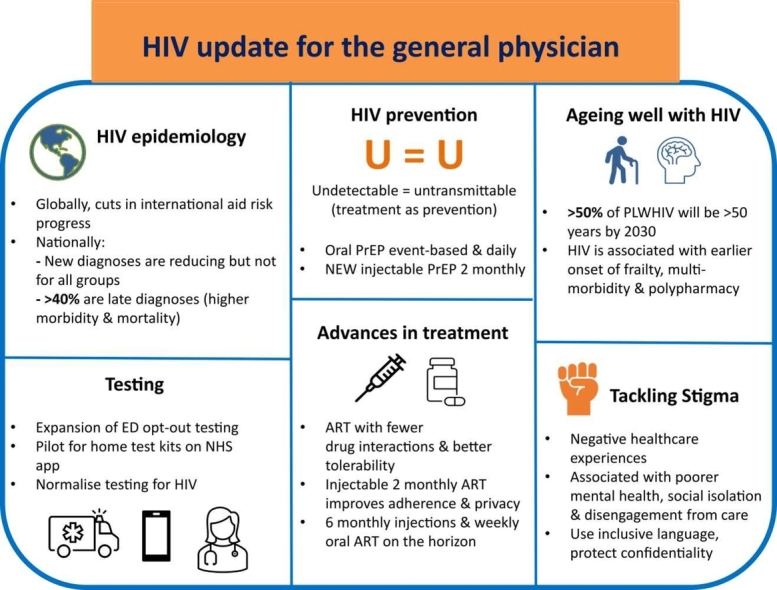

## Global and national changes in the epidemiology of HIV

Globally, new HIV infections peaked in the late 1990s and have been declining since, largely due to the introduction of effective antiretroviral therapy (ART). However, there were still an estimated 1.3 million new HIV infections worldwide in 2024. For the first time since the start of the epidemic, most new infections occurred outside sub-Saharan Africa. The region has seen substantial progress: compared with 2010, new infections in 2024 were 56% lower in eastern and southern Africa and 55% lower in western and central Africa. In contrast, other regions have seen increases, including a 94% rise in the Middle East and North Africa, a 7% rise in Eastern Europe and Central Asia, and a 13% rise in Latin America. These trends risk being made worse by reductions in international HIV funding.^1^

In the UK, there were 3,043 new HIV diagnoses in 2024, a 4% decrease from 3,169 in 2023. In England, there were 2,773 new diagnoses in 2024, a 2% decrease from 2023, but an increase of 24% from 2,240 in 2020. Opt-out testing for blood-borne viruses in emergency departments contributed to 8% of all new diagnosis in 2024.^2^

Of newly diagnosed people with HIV in England, 29% were gay and bisexual men, 23% were heterosexual men, and 27% were heterosexual women. Diagnoses among gay and bisexual men decreased by 6% (859 in 2023 to 810) with the decrease largest among White men compared to men from ethnic minorities, who constituted 35% of all new diagnoses in gay and bisexual men in 2024. 60% of people newly diagnosed with HIV in England were among people born abroad (1,669 of 2,773).^2^

Among heterosexual men, new diagnoses increased by 3% between 2023 and 2024 (615 to 634), while there was a 1% decrease among heterosexual women. Over the same period, diagnoses increased among Black African men by 15% (231 to 265), Black Caribbean men by 44% (16 to 23) and men with other or mixed ethnicity by 2% (43 to 44). Systemic barriers to pre-exposure prophylaxis (PrEP), stigma hindering testing, disproportionate numbers of late diagnoses, socioeconomic inequalities, and the failure of accurate health information to reach underserved communities in the UK all contribute to the increases that we are seeing in new HIV diagnoses in these groups. Compared to White gay and bisexual men who have sex with men (MSM), other groups are less likely to have their need for PrEP identified and, where it is identified, are less likely to initiate PrEP.^2^

Late diagnosis remains a major problem. In 2024, over 40% of new diagnoses in England were late (CD4 count <350 cells/mm³). Older adults were disproportionately affected by late diagnoses (HIV was diagnosed late in 55% of those diagnosed aged 50–64 years old, and 61% of those aged 65 years and over). Data on late diagnoses show that HIV was diagnosed late predominantly in Black African heterosexual men (57% of diagnoses were late) and other ethnic minority heterosexual adults (49% of diagnoses were late).^2^ In 2023, people diagnosed late were 10 times more likely to die within a year compared to those diagnosed earlier. Starting ART immediately after diagnosis, regardless of CD4 count, significantly reduces illness and death.^3^, ^4^

## Updates in HIV prevention

Preventing HIV transmission requires a multifaceted approach, including condom use, sexual health education, pre- and post-exposure prophylaxis, treatment as prevention, needle exchange programmes and routine HIV testing.

Treatment as prevention refers to the impact of viral suppression on transmission risk. People living with HIV (PLWH) who maintain an undetectable viral load (<200 copies/mL) have zero risk of passing on HIV through sex even without condoms, a principle summarised as U=U (undetectable=untransmittable).^5^, ^6^, ^7^, ^8^ In practice, this means that most people living with HIV not only have good health outcomes, but also cannot transmit the virus sexually. In 2015, WHO guidelines changed, advising that people start ART as soon as possible after diagnosis, both for individual health benefits and because early treatment reduces onward transmission. The immense efforts to diagnose and provide timely treatment for people living with HIV have contributed to an overall decline in the number of new infections and AIDS-related deaths globally.^1^

Post-exposure prophylaxis (PEP) has been available since the 1990s. It must be initiated within 72 h of a high-risk exposure and taken for 28 days. In the UK, PEP is available in sexual health clinics and emergency departments. Although robust trial data are limited, PEP is widely accepted as an effective intervention. People prescribed PEP for non-occupational exposures should be offered PrEP once they complete the course.^9^

HIV pre-exposure prophylaxis (PrEP) has transformed prevention over the past decade. Daily oral PrEP with tenofovir disoproxil fumarate (TDF) was endorsed by the WHO in 2012 and approved by NICE in 2021. When taken correctly, PrEP reduces HIV risk by almost 100%. Event-based dosing is also effective: for anal sex, two tablets are taken at least 2 hours before sex, followed by one daily for 48 h afterwards; for vaginal, neovaginal or injecting-related risks, the loading dose is followed by once daily dosing for 7 days. Tenofovir alafenamide (TAF)-based PrEP is a safer alternative for bone and kidney health and is preferred in individuals with osteoporosis or kidney disease, or those under 18 (requires MDT approval).^10^

Injectable PrEP is an important development. Two trials published in 2021 showed that 2-monthly cabotegravir injections were more effective than oral TDF-based PrEP.^11^, ^12^ In 2025, NHS Scotland and NICE approved cabotegravir injections for people who are unable to take oral PrEP either because they cannot swallow tablets, have medical contraindications or for social reasons. More recently, 6-monthly lenacapavir injections reduced HIV acquisition by 96–100% in trials among adolescent girls, young women and MSM, though cost and regulatory approval remain barriers.^13^, ^14^ If affordable, injectable PrEP could have a significant impact on the global epidemic.

Multipurpose prevention technologies are also being developed. The dual prevention pill, which combines oral contraception with PrEP, may reduce stigma and improve access among women, a group underserved by HIV prevention efforts.^15^

Despite these advances, PrEP uptake is far below global targets. In 2024, around 3.9 million people accessed oral PrEP, up from 200,000 in 2017, but well short of the UNAIDS target of 21.2 million by 2025.^1^ In the UK, the 2025 BASHH PrEP guidelines highlight inequities in access and recommend strategies to reach underserved communities.^10^ Expanding PrEP delivery will be critical to achieving the UK government’s target of zero new HIV transmissions by 2030.^16^

Given the high rates of late diagnosis, HIV testing remains a cornerstone of prevention. Earlier diagnosis reduces morbidity, mortality and transmission. Some of the biggest barriers to HIV testing come from within healthcare settings, including clinicians’ assumptions about risk or reluctance to offer a test. Testing should be offered widely, and without stigma or unnecessary barriers such as pre-test counselling. A routine approach, similar to testing for other chronic conditions, helps to normalise testing, and ensures that people are diagnosed early. Since 2022, opt-out blood-borne virus testing has been implemented in emergency departments in high (≥2 per 1,000 HIV prevalence) and extremely high (>5 per 1,000) prevalence areas of England, recently expanding to 47 further sites. This programme is identifying both new diagnoses and individuals who have disengaged from care.^2^, ^17^ Testing people who present with HIV indicator conditions continues to be essential everywhere, but is particularly important to remember in lower prevalence areas where opt-out testing is not happening.^18^

## Updates in antiretroviral therapy

The development of newer antiretroviral therapies (ART) has markedly improved tolerability, simplified regimens and reduced the frequency of clinically significant drug–drug interactions (DDIs). Nevertheless, interactions remain an important consideration in general practice. They can occur in both directions: concomitant medications may alter ART levels, while antiretroviral drugs can influence the metabolism of commonly prescribed agents for comorbidities. Most interactions are manageable, often through dose adjustments when boosting agents such as ritonavir or cobicistat are used. However, serious complications can occur. For example, boosted protease inhibitors can markedly increase systemic steroid exposure even when non-oral routes (eg inhaled, injected, intranasal) are used, resulting in iatrogenic Cushing’s syndrome and adrenal suppression. Boosted protease inhibitors also interact with statins, in most cases atorvastatin can still be given but starting at a lower dose with a lower maximum safe dose, alongside increased vigilance for symptoms of toxicity. Some co-medications can significantly reduce the efficacy of certain antiretrovirals, examples of significant co-prescriptions to avoid include oral rilpivirine and proton pump inhibitors; also commonly prescribed oral integrase strand transfer inhibitors (eg bictegravir in the combined tablet known as ‘biktarvy’, dolutegravir, raltegravir) can interact with cations including oral iron, multivitamins, antacids containing magnesium or aluminium and calcium supplements. Clinicians should remain vigilant and always check for potential interactions using the ‘Liverpool HIV Drug Interactions Checker’ or seek pharmacist advice where uncertainty exists.^19^

Another clinically relevant issue is the increasing use of GLP-1 receptor agonists for diabetes and obesity management. Current evidence suggests a potential pharmacokinetic interaction with oral rilpivirine, an antiretroviral agent used in oral regimens as well as the long-acting injectable regimen cabotegravir/rilpivirine if oral lead-in is required, and for oral ʻbridging therapy’ if injections are delayed: GLP-1 receptor agonists slow gastric emptying, which may impair the absorption and reduce the bioavailability of oral rilpivirine. The clinical impact is uncertain, but caution is advised and HIV specialists should be consulted before co-prescribing these agents, as the antiretroviral regimen may need to be changed first.^20^

Integrase strand transfer inhibitors (INSTIs), such as dolutegravir and bictegravir, now form the backbone of most first-line regimens in the UK.^21^ These agents are generally well tolerated, available in once-daily single-tablet combinations, have a high barrier to resistance, and are associated with fewer DDIs compared with non-nucleoside reverse transcriptase inhibitors (NNRTIs) or protease inhibitors. However, emerging evidence has highlighted potential metabolic effects. Weight gain, sometimes substantial, has been observed following initiation or switching to INSTI-based regimens, particularly when used in combination with tenofovir alafenamide (TAF).^22^, ^23^ Observational data also suggest an increased risk of diabetes in some populations, although the evidence is conflicting and research is ongoing.^24^ While specialist HIV services routinely monitor weight and metabolic parameters, clinicians should be alert to rapid weight increases or incident diabetes in people living with HIV who recently commenced or switched to these regimens.

Another key advance is the introduction of long-acting injectable ART. The first licensed regimen in the UK (cabotegravir and rilpivirine), is administered intramuscularly every 2 months.^25^ This approach is particularly suitable for individuals who struggle with daily oral therapy due to stigma, pill fatigue or cognitive difficulties. Early experience has shown high levels of acceptability and effectiveness when delivered through specialist HIV clinics.^26^, ^27^ Additional long-acting options are in development, including 6-monthly subcutaneous formulations (eg lenacapavir) and investigational weekly oral regimens.^28^ These innovations have the potential to transform HIV care and adherence, although careful attention to timely dosing and follow-up remains essential.

## Ageing and HIV

Thanks to the success of antiretroviral therapy, people living with HIV are now living well into older age. By 2030, it is estimated that more than half of those receiving HIV care in the UK will be over the age of 50.^29^ As this population ages, comorbidities become increasingly important. There is some evidence that PLWH experience age-related conditions earlier than the general population, including cardiovascular disease (CVD), osteoporosis and frailty conditions. PLWH are more likely to have multiple comorbidities at an earlier age, with the associated risks of polypharmacy and negative impact on quality of life.^30^, ^31^

The reasons for this earlier onset are multifactorial. They reflect not only HIV-specific factors such as persistent immune activation despite viral suppression and the long-term impact of earlier, more toxic antiretroviral regimens, but also broader social determinants of health including socioeconomic disadvantage, stigma and variable access to healthcare. We may also be observing a legacy effect, as the cohort of PLWH who lived through the early years of the AIDS pandemic are now ageing. They are more likely to have had a delayed start of treatment and longer periods with unsuppressed viral loads with associated inflammation. Cardiovascular complications are a particular concern. People with HIV have higher rates of atherosclerosis and ischaemic heart disease, leading to myocardial infarction and cerebrovascular disease at younger ages. Importantly, commonly used cardiovascular risk scores such as QRISK3 underestimate risk in people living with HIV because they do not account for HIV infection, which is itself associated with increased CVD and mortality even when well controlled.^32^

Frailty is another major issue. Geriatric syndromes, including frailty, occur on average a decade earlier in people with HIV compared with the general population. This can create challenges, as many people may appear too young to access geriatric services, despite having equivalent needs. Some studies have shown that PLWH are more likely to be diagnosed with cognitive impairment, although more research is needed, and established risk factors for cognitive impairment such as vascular disease, diabetes, smoking and depression are common in PLWH.^33^ Early recognition and modification of risk factors is essential, but symptoms may be subtle and overlap with depression or treatment side effects.

Osteoporosis is more common in people with HIV and is compounded by HIV-specific risk factors. These include prolonged exposure to certain antiretrovirals such as tenofovir disoproxil fumarate, as well as the effects of menopause in women. Standard fracture risk scores such as FRAX may underestimate fracture risk in this population, as HIV is not included as a variable. As a result, HIV services in the UK assess all people over 50 for fragility fracture risk and determine the need for DEXA scanning accordingly.^34^

Preventive pharmacotherapy is playing an increasing role. The multinational REPRIEVE trial enrolled more than 7,700 people with HIV aged 40–75 with low-to-moderate traditional CVD risk scores (median 4.5%). It demonstrated that daily pitavastatin reduced the risk of major adverse cardiovascular events by 35% compared with placebo over 5 years.^35^ Following these results, BHIVA released new guidance in 2024 recommending that all people with HIV aged ≥40 are counselled on the trial findings and offered statin therapy, regardless of their calculated cardiovascular risk or lipid profile.^36^ Coordinated care between HIV specialists and GPs is critical to ensure effective implementation and safe prescribing, including DDI checks.

In summary, ageing with HIV presents unique challenges. Comorbidities occur earlier, frailty is more common, and cognitive and cardiovascular risks are heightened.^33^, ^35^ Osteoporosis and fracture risk require particular attention. While HIV services already perform targeted screening, effective management requires close collaboration with GPs, hospital specialists and allied health professionals to integrate preventive care, optimise screening, be vigilant for polypharmacy, and address the broader determinants of health that influence ageing in this population.

## Health promotion and holistic care in people with HIV

This section outlines specific health promotion interventions for people living with HIV, including information about family planning, breastfeeding and the menopause.

BHIVA recommends that PLWH are offered some key vaccinations. Human papillomavirus (HPV) vaccinations are recommended up until 45 years for all MSM regardless of HIV status, and up until 40 years for women living with HIV. HPV infection persists for longer in PLWH, and is associated with an increased risk of vaginal, cervical and anal intra-epithelial neoplasia progressing to invasive cancer. The PCV-13 pneumococcal vaccine is recommended for people living with HIV because of the increased risk of invasive pneumococcal disease and associated mortality. Hepatitis A, B and C status is checked at the time of new HIV diagnosis and vaccination offered if non-immune to hepatitis A or B. This is because PLWH are more likely to develop chronic hepatitis B, which is associated with a worse prognosis, including increased risk of hepatocellular carcinoma. Annual flu vaccines and COVID boosters are also advised. MMR and chickenpox replicating vaccinations are recommended but require CD4 counts >200 mm^3^, and there are post-exposure recommendations for both measles and varicella in those with lower CD4 counts or those who are measles/varicella seronegative.^37^ Shingrix, a non-live vaccine for shingles, has replaced Zostavax in the UK immunisation programme. As it is not a live vaccine, it can be given if the CD4 count is <200, and is also safe to give in people who are non-immune to VZV. Current BHIVA guidance advises that Shingrix is offered to PLWH at the same age as the general population, unless they have a CD4 <200, or they have other reasons to be severely immunosuppressed, in which case it can be given earlier (if a person’s CD4 is <200, The Green Book advises that Shingrix can be given from the age of 18 years, superseding the BHIVA position statement on shingles from 2023).^38^, ^39^ Updated BHIVA vaccine guidelines will be published in 2026; these are expected to significantly lower the age thresholds for the shingles vaccine in PLWH, including in those with higher CD4 counts. BCG tuberculosis vaccine and live attenuated typhoid vaccines are contraindicated. Please refer to the BHIVA vaccine guidelines for further information, including vaccine safety and efficacy based on CD4 count.^40^

Cancer screening in PLWH is the same as in the general population (faecal immunochemical test for bowel cancer and mammograms for breast cancer) except for cervical screening, which is yearly for PLWH rather than every 5 years.^34^

Reproductive health and family planning is a core part of supporting people living with HIV to live fulfilled lives. The full range of contraceptive options should be available to those living with HIV who do not wish to conceive (check for DDI before prescribing).^19^ For those planning to conceive, no additional measures are required to prevent transmission if the person has an undetectable viral load on ART. Where the person has detectable viraemia, HIV-PrEP can be taken by partners to prevent transmission. A hard-fought-for change in law was made last year, finally allowing people living with HIV who have been on ART for at least 6 months with an undetectable viral load to donate their sperm or eggs to someone they know.^41^ This marks a long-overdue move towards equality in reproductive rights for people with HIV in the UK. Vertical transmission of HIV is now very rare in the UK due to effective antenatal screening, with transmission rates of 1 in 1,000 where the pregnant person is receiving ART in pregnancy and is undetectable (<50 copies/mL) by 36 weeks.^42^ Formula feeding is still recommended in preference to breast/chest-feeding because it carries zero risk of transmission (breast/chest-feeding has a very low transmission risk). BHIVA provides clear guidance on feeding newborns to enable parents to make an informed decision, including advice on how to minimise risk if breast/chest-feeding is chosen.^42^, ^43^

As our population living with HIV in the UK is ageing, the proportion of women living with HIV going through the menopause has increased. Women with HIV are more likely to have osteoporosis, cardiovascular disease, and more prominent vasomotor and psychological symptoms of the menopause. Evidence also shows that the menopause negatively impacts adherence to ART and attendance at HIV clinics, and a number of HIV services now run specialist clinics for women to better address their needs.^44^

## Stigma

Despite major advances in treatment and prevention, stigma remains one of the biggest barriers to effective HIV care. Stigma influences how people perceive their condition, whether they share their status, and how readily they access services. It is strongly associated with poorer mental health, social isolation and disengagement from care.^45^ For healthcare professionals, it is important to remember that every interaction can shape how a person feels about seeking help in the future.

Patients’ experiences when accessing healthcare are critical. Many PLWH report negative encounters in healthcare settings, including breaches of confidentiality, inappropriate comments, or unnecessary infection-control precautions.^46^ Such experiences can reinforce feelings of shame or difference, and may deter people from attending again. For some, stigma is compounded by other factors such as ethnicity, gender, sexuality or migration status, which can intersect to create additional barriers.^47^

In clinical practice, stigma can undermine prevention as well as treatment. People may delay HIV testing because they fear being judged, or avoid discussing sexual health, substance use or mental health concerns. Stigma can also discourage uptake of PrEP and PEP, particularly among women, migrants and older people, who may not see these interventions as ʻfor them’.^47^ Addressing stigma is therefore integral to achieving the government’s target of ending new HIV transmissions by 2030.

General practice, other frontline services and non-HIV specialists in secondary care have an important role. The language used in consultations, the visible inclusivity of the clinic environment, and the way that confidentiality is handled all matter. Small changes such as normalising HIV testing as part of routine care, using non-judgemental language and being open to conversations about sexual health can help people feel respected and supported.

## Conclusions

HIV has been transformed from a fatal illness into a manageable chronic condition, with life expectancy for PLWH approaching that of the general population. This success reflects the effectiveness of modern ART and the dedication of specialist services. Yet, important challenges remain. Rising numbers of new diagnoses and persistently high rates of late diagnosis in certain groups highlights inequity in access to HIV prevention and testing.

**Fig. 1:**
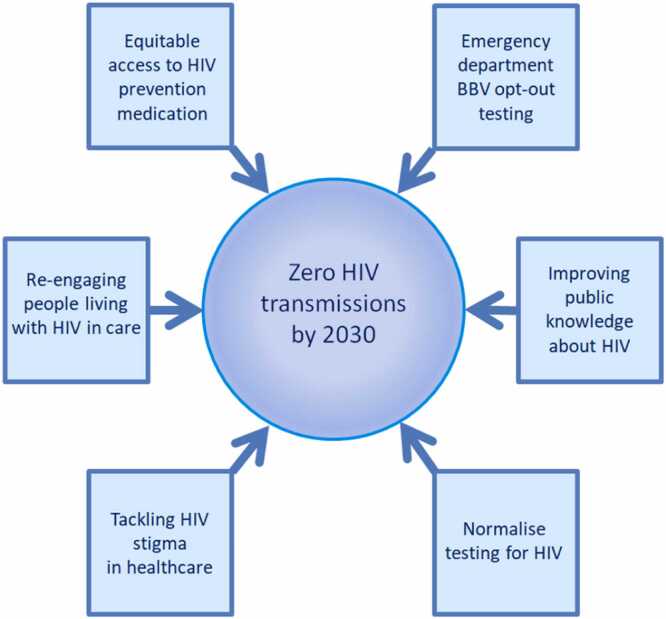
Working towards zero new HIV transmissions by 2030.

The announcement of the updated HIV Action Plan in December 2025 shows a renewed focus on ending HIV transmission by 2030, and is supported by the biggest new investment in years.^16^ The plan prioritises equitable access to HIV prevention services, focusing on under-served communities and retention in care after diagnosis, expands opt-out testing, plans to pilot home HIV testing via the NHS app and addresses stigma (see Fig. 1).

For all non-HIV specialist clinicians, remember that testing should be offered as a normal part of clinical care, without unnecessary barriers, to ensure timely diagnosis and early treatment, and that stigma continues to undermine prevention, diagnosis and treatment. Disengagement and loss to follow-up is an area that requires renewed and intense focus; non-HIV specialist clinicians are crucial in helping to identify disengaged patients through testing, asking about active HIV care and treatment, and facilitating the rapid re-referral of disengaged people who attend other healthcare services. For general practitioners, alongside stigma-free testing, there is the additional opportunity to promote PrEP in the wider community. This is critical for reaching people who do not access sexual health services due to stigma.

Every healthcare interaction offers an opportunity either to reinforce or reduce stigma. By using inclusive language, ensuring confidentiality and approaching HIV as we would any other chronic condition, healthcare professionals can help create an environment in which patients feel respected, supported and empowered to engage with care.

## CRediT authorship contribution statement

**Jaime H. Vera:** Writing – review & editing, Writing – original draft, Supervision, Conceptualization. **Miriam Ringshall:** Writing – review & editing, Writing – original draft, Conceptualization.

## Funding

This research did not receive any specific grant from funding agencies in the public, commercial, or not-for-profit sectors.

## Declaration of competing interest

The authors declare that they have no known competing financial interests or personal relationships that could have appeared to influence the work reported in this paper.
